# Point of care colourimetric and lateral flow LAMP assay for the detection of *Haemonchus contortus* in ruminant faecal samples

**DOI:** 10.1051/parasite/2021078

**Published:** 2021-12-15

**Authors:** Rojesh Khangembam, Mariann Tóth, Nóra Vass, Marián Várady, Levente Czeglédi, Róbert Farkas, Alistair Antonopoulos

**Affiliations:** 1 Department of Animal Science, Institute of Animal Science, Biotechnology and Nature Conservation, Faculty of Agricultural and Food Sciences and Environmental Management, Böszörményi ut. 138, University of Debrecen Debrecen 4032 Hungary; 2 Department of Animal Science, Institute for Agricultural Research and Educational Farm, Faculty of Agricultural and Food Sciences and Environmental Management, Böszörményi ut. 138, University of Debrecen Debrecen 4032 Hungary; 3 Doctoral School of Animal Science, University of Debrecen Debrecen 4032 Hungary; 4 Institute of Parasitology, Slovak Academy of Sciences, Hlinkova 3 04001 Košice Slovakia; 5 Department of Parasitology and Zoology, University of Veterinary Medicine István u. 2 Budapest 1078 Hungary; 6 School of Veterinary Medicine, College of Medical, Veterinary and Life Sciences, Garscube Campus Bearsden Road, University of Glasgow Glasgow G61 1QH Scotland

**Keywords:** LAMP assay, Lateral flow, *Haemonchus contortus*, Point-of-Care Diagnosis, Fill-FLOTAC^®^

## Abstract

In this study, we present an optimised colourimetric and a lateral flow LAMP assay for the detection of *Haemonchus contortus* in small ruminant faecal samples. Using a previously published LAMP primer set, we made use of commercially available colourimetric LAMP and lateral flow kits and combined this into an optimised diagnostic assay which was then tested on field faecal samples from Eastern and South-Eastern Hungary as well as a pure *H. contortus* egg faecal sample from Košice, Slovakia. Both assays showed no conflicts in visual detection of the results. Additionally, we modified and tested several centrifuge-free DNA extraction methods and one bead-beating egg lysis DNA extraction method to develop a true point of care protocol, as the source of the starting DNA is the main rate-limiting step in farm-level molecular diagnosis. Out of the various methods trialed, promising results were obtained with the magnetic bead extraction method. Sample solutions from the Fill-FLOTAC^®^ technique were also utilised, which demonstrated that it could be efficiently adapted for field-level egg concentration to extract DNA. This proof of concept study showed that isothermal amplification technologies with a colourimetric detection or when combined with a lateral flow assay could be an important step for a true point of care molecular diagnostic assay for *H. contortus*.

## Abstract (fr)

Dans cette étude, nous présentons un test colorimétrique optimisé et un test LAMP à flux latéral pour la détection d’*Haemonchus contortus* dans des échantillons de selles de petits ruminants. À l’aide d’un ensemble d’amorces LAMP publié précédemment, nous avons utilisé des kits colorimétriques LAMP à flux latéral disponibles dans le commerce et les avons combinés dans un test de diagnostic optimisé qui a ensuite été testé sur des échantillons de matières fécales de terrain provenant de l’est et du sud-est de la Hongrie ainsi que d’un échantillon d’œufs de *H. contortus* provenant de selles de Košice, Slovaquie. Les deux tests n’ont montré aucun conflit dans la détection visuelle des résultats. De plus, nous avons modifié et testé plusieurs méthodes d’extraction d’ADN sans centrifugation et une méthode d’extraction de l’ADN des œufs par lyse par billes pour développer un véritable protocole de point d’intervention, car la source d’ADN de départ est la principale étape limitante du diagnostic moléculaire au niveau de la ferme. Parmi les différentes méthodes testées, des résultats prometteurs ont été obtenus avec la méthode d’extraction par billes magnétiques. Des solutions d’échantillons de la technique Fill-FLOTAC^®^ ont également été utilisées, ce qui a démontré qu’elle pouvait être efficacement adaptée à la concentration d’œufs sur le terrain pour extraire l’ADN. Cette étude de preuve de concept a montré que les technologies d’amplification isotherme avec une détection colorimétrique ou lorsqu’elles sont combinées avec un test de flux latéral pourraient être une étape importante pour un véritable test de diagnostic moléculaire au point d’intervention pour *H. contortus*.

## Introduction

*Haemonchus contortus* (Rudolphi, 1803) Cobb, 1898 is a haematophagous gastrointestinal nematode (GIN) parasite of small ruminants [3]. *Haemonchus contortus* is one of the most pathogenic GIN parasites and has the potential to cause high mortality rates and significant production losses if left untreated or unmanaged [25, 34]. Estimates from the sheep farming sector in Australia placed *H. contortus* as one of the leading causes of production loss [15]. Globally, haemonchosis is responsible for significant production losses due to reduced milk output, growth rates, and meat yield [3, 25]. *Haemonchus contortus* is now established worldwide across a wide range of climatic zones [3]. Blood feeding stages are responsible for the pathology of haemonchosis and its consequent anaemia [51] with an adult worm able to consume ~30–50 μL of blood daily [6]. *Haemonchus contortus* shows remarkable fecundity, with a single female able to shed upwards of 15,000 eggs per day [15], has an establishment rate of ~60% [12], and has one of the shortest patency periods of any GIN (~15 days) [15].

The current diagnosis of GIN infection relies heavily on microscopy-based methods examining the presence of eggs in stools in both veterinary [7, 23, 49, 53] and human medicine [5, 22]. The picture is complicated by the cumulative effects of mixed *Strongyle* worm infection, common in livestock [9, 41]. Except for *Nematodirus*, eggs of the remaining *Strongyle* genera are hard to distinguish under the microscope. Species-level identification commonly relies on time-consuming larval culture, taking ~7–10 days. Currently, a commercial lectin staining kit is available for the identification of *H. contortus* eggs [41]. Faecal Egg Counting (FEC) microscopy techniques employed in the detection of trichostrongyle/*Strongyle* eggs commonly use variations of the McMaster technique, which is the WAAVP recommended method for veterinary GIN diagnosis [7, 8, 40]. FLOTAC^®^ represents an improvement in microscopy-based flotation techniques by integrating the flotation chamber and counting chamber into a single device [10, 29, 52], streamlining the faecal egg counting procedure, and is effective in detecting the presence of *Strongyle* GIN infection but cannot be used effectively for species-specific identification. However, a study has claimed to detect a few GIN parasite species in howler monkeys using Mini-FLOTAC^®^/FLOTAC^®^ in field conditions [1]. Yet, the technique still relies on centrifugation and can result in reduced egg counts, and lacks 100% sensitivity [17, 19, 36]. Mini-FLOTAC^®^ builds on the FLOTAC^®^ protocol wherein faeces and floatation solution are homogenised then loaded into flotation chambers present in a reading disk. When combined with the Fill-FLOTAC^®^ system, it can present in one package the advantageous combination of weighing, homogenisation, filtration and counting-chamber filling [1, 11]. Despite improvements, there remain several drawbacks to established faecal egg counting techniques, including significant variation in eggs per sample due to aggregation and non-random distribution through the sample and daily fluctuations in egg output [28–30]. Microscopy based techniques are also heavily dependent on the competence of the staff, and the quality of the microscope [42]. As such, it is of use to develop improvements for confirmatory species-specific diagnosis by integrating molecular diagnostics into established diagnostic protocols.

Molecular diagnosis of *H. contortus* poses a unique set of challenges when designing tests. *Haemonchus contortus* eggs are typically used both in the coprological microscopic analysis [7, 8] and molecular testing [4, 33, 45]. *Haemonchus contortus* eggs are small, durable and resistant presenting a challenge both to extracting and acquiring a sufficient quantity of genetic material [13]. In addition, faeces are problematic as a medium for direct detection due to copious Polymerase Chain Reaction (PCR) inhibitor molecules [21]. PCR tests have been developed for the detection of *Strongyle* eggs [4, 43–45]; however, due to the requirement for sample processing and thermocycling equipment, these remain restricted to well-supplied reference laboratories and are not suitable for field deployment. Nonetheless, PCR remains the gold standard and it is still in use for species-specific confirmatory tests.

Loop-mediated Isothermal Amplification (LAMP) is an isothermal nucleic acid amplification test that uses a *Bst* polymerase combining optimal temperature of 60–65 °C with inherent strand displacement activity, allowing the elimination of thermocycling [38]. LAMP makes use of two essential primer pairs and one optional primer pair to increase reaction speed [35, 38]. LAMP has been successfully demonstrated for *H. contortus* detection [33]. LAMP is ideally suited to point of care (POC) and direct detection applications as it is comparatively more tolerant of PCR inhibitor molecules found in blood or faeces [21]. LAMP is also tolerant of multiple primer sets used simultaneously and can thus be readily multiplexed. This has been successfully demonstrated for important human helminth *Taenia* spp. [37] although to date, this is the only multiplexed helminth LAMP developed. However, numerous examples exist of multiplexed LAMP assays demonstrated to date in other pathogen groups [31].

LAMP is amenable to a variety of endpoint detection methods. One particular note for point of care applications is convenient and fast result interpretation. Both colourimetric detection and lateral flow have the advantage of requiring minimal equipment and ease of interpretation with the naked eye. Colourimetric LAMP makes use of colour changing dyes in response to DNA polymerisation and has been successfully demonstrated for a range of human and veterinary pathogens [26, 47, 48, 50]. Lateral Flow (LF) is a well-established paper-based platform for the detection of analytes in complex mixtures using an antibody-antigen capture system. It is widely used in both hospital and field settings [24]. It can be readily adapted to detect amplified DNA by tagging primers [20, 46]. LF is cheap to produce, easy to use with simple naked eye interpretation of readouts. The technique has been widely tested and demonstrated across many pathogens and starting materials [24], besides being easily amenable to multiplexing [16]. LAMP assays are also amenable to quantification based diagnosis at the point of care, as has been demonstrated with smartphone-based technologies utilising colourimetric detection [54].

In this study, we built on the previously published *H. contortus* LAMP assay [33]. Herein, we used a primer set published in Melville et al. (2014) [33] but adapted and optimised it to a colourimetric naked-eye detection system of *H. contortus* in field samples. We also present an optimised LF-LAMP assay using tagged primers as a proof of concept step towards the future development of a POC multiplexable veterinary GIN LF-LAMP assay. Finally, we also compared various methods to obtain DNA by eliminating the use of the standard laboratory high speed centrifuge machine keeping in view the POC settings as obtaining the starting DNA template is an important aspect for a POC diagnosis. By using the Fill-FLOTAC^®^ system along with a set of portable centrifugation equipment, we have also integrated the egg concentration step (from the Fill-FLOTAC^®^ solution) to be utilised for DNA extraction. Thus, we present a proof of concept study on POC Colourimetric/LF-LAMP assay for the detection of *H. contortus* with the potential to be multiplexed in the future that could offer confirmatory speciation diagnosis.

## Materials and methods

### Ethics statement

The faecal samples used were collected from the animals as per the Hungarian Animal Protection and Welfare Act (Act XXVIII of 1998, 3.§). For the *H. contortus* isolate MHCo3 (ISE), L3 were fed orally to the lambs; the Ethics Committee of the Institute of Parasitology of the Slovak Academy of Sciences gave approval for animal use and experiments under European Union guidelines (EU Directive 2010/63/EU for animal experiments).

### Study location and sample processing

The practical experiments of this proof of concept study were conducted at the Institute of Animal Science, University of Debrecen, Hungary with the main concept of the study and initial assay design being established at the School of Veterinary Medicine, University of Glasgow, Scotland as part of a collaboration. A pure *H. contortus* eggs culture (designated herein as PEK), maintained by passage through two worm-free 5–6-months old merino lambs infected orally with approximately 5000 L3 infective larvae of susceptible isolate, MHCo3 (ISE), was also obtained from the Institute of Parasitology, Košice, Slovakia from which gDNA was extracted. Genomic DNA samples (designated herein as PCB) were also provided by the Department of Parasitology and Zoology, University of Veterinary Medicine, Budapest. The two above-mentioned gDNA served as the positive control template for the molecular assays performed in this study.

All the field samples used were obtained from another routine faecal egg count (FEC) survey study (Toth et al, unpublished) on various sheep farms (assigned as Farm I–X) in Eastern and South-Eastern Hungary. These faecal samples were collected from randomly selected individual sheep as per the guidelines laid down in [7] with slight modifications. FEC was done using saturated salt solution (specific gravity of 1.200; dilution ratio of 1:10) as the floatation medium, using the Mini-FLOTAC^®^ along with the Fill-FLOTAC^®^ system following the protocols in [11]. Additionally, suspected adult *H. contortus* obtained from farmed deer abomasums and also a pooled goat faecal sample were also utilised (assigned as Farm DF and Farm GF, respectively) in the course of the optimised assay trials. The samples used during the initial optimisation stages as well as the subsequent trials of the optimised assays are summarized in Table 1.

**Table 1:** Average EPG of the farms (*n* > 25 sheep per farm) used in the respective assay designs.

Farm ID	Average EPG	Result of Assay Used				Remarks
		LAMP Optimisation	LF Assay Optimisation	Optimised LAMP	PCR	
Farm I	600	✓	NA	✓	✓	
Farm II	150*	*✖	NA	✓	✓	*✖**:** pooled exclusive of samples with suspected trichostrongyle eggs
Farm III	276.47	✓	NA	✓	✓	
Farm IV	982.22	NA	✓	✓	✓	
Farm V	1750	NA	✓	✓	✓	
Farm VI	5.76	NA	✖	✖	✖	
Farm VII	113.70	NA	✓	✓	✓	
Farm VIII	767.20	NA	✓	✓	✓	
Farm IX	168	NA	✓	✓	✓	
Farm X	–	NA	NA	✓	✓	
Farm DF**	–	✓	NA	✓	✓	
Farm GF***	–	½	NA	✓	✓	½: One weak positive

*Only three individual sheep had trichostrongyle egg counts. **Adult worms only. *** Pooled faecal sample submitted by the farmer. NA: Not applicable as it was not used during the optimisation. ✓**:** Positive result. ✖**:** Negative result.

### DNA extraction using commercial kit

Those individuals faecal samples having Egg Per Gram (EPG) ≥ 100 were separately stored in airtight Ziplock pouches at −4 °C for further DNA extraction. Another farm with a low egg count and no visually detected trichostrongyle eggs (Farm VI; EPG = 5.76) was also used. This DNA extraction was performed from both the pooled as well as individual samples, which were to be utilised during the assay optimisation and trials. For the pooled sample DNA extraction, a minimum of five individual animals was taken into account. DNA from all the faecal samples was extracted using a QIAamp Fast DNA Stool Mini Kit (Qiagen), following the manufacturer’s instructions, and DNA from the adult worms from Farm DF was extracted using an E.Z.N.A.^*®*^ Tissue DNA Kit (Omega Bio-Tek Inc.)*.*

### LAMP assay

A previously validated primer set published in Melville et al. (2014) [33] (detailed in Table 2) was used for this assay. For detection of the result visually, a WarmStart^®^ Colorimetric LAMP 2X Master Mix (DNA & RNA) kit (New England Biolabs Ltd, UK) was used. The final LAMP reaction primer mix contained: 1.6 μM of each FIP and BIP primers, 0.8 μM of each Loop Forward and Loop Backward primers and 0.2 μM of each F3 and B3 primer. LAMP assay was carried out (as per the kit manual) in 25 μL reaction volume consisting of 12.5 μL of WarmStart^®^ Colorimetric LAMP 2X Master Mix, 2.5 μL of the primer mix, 1 μL of template DNA and 9 μL of molecular grade water (AccuGene). The reaction was run using an Alpha Cycler 1 (PCRmax, UK) at temperatures of 60 °C, 61 °C, 62 °C and 63 °C for 30–45 min using the positive DNA control as well as a non-template control (NTC) during optimisation. Once this initial optimisation was established, the assay was replicated successfully using the VWR^®^ Advanced Mini Dry Block Heaters (VWR International) at 62 °C for 30 min using the positive control templates. A 10-fold serial dilution of the positive template DNA was also prepared, and 1 μL each of the dilutions was subsequently used for the LAMP reaction at 62 °C for 30 min to test analytical sensitivity.

**Table 2:** LAMP and PCR primer sequences used.

Primer	Sequence (5′–3′)
FIP*	AACAATCACAGCCGCCACTAAGCTCTATTACATGAGGTGTC
BIP*	TCATTGATGGTTGAGCTTGAGACTTGTTCGTACTTAACCACCATCA
F3	GGTTCCATTGATCACGAGAA
B3	CAGTACACCACATACTCAAGAA
FLP	AAGCGGCTCATGTCATACAT
BLP	CTATAATACTGCCTCGCCGTT
PCR Primers	Sequence (5′–3′)
Forward	GTTACAATTTCATAACATCACGT
Reverse	TTTACAGTTTGCAGAACTTA

*The same sequences were tagged with FITC and BIO to get tagged FIP and BIP, respectively for the LF-LAMP Assay.

### Lateral flow assay

A HybriDetect -Universal Lateral Flow Assay Kit (Milenia Biotec GmbH, Germany) was used for endpoint detection of dual tagged LAMP amplicons. LAMP primers described in [33] were tagged with Fluorescein isothiocyanate (FITC) and biotin (BIO) at the 5′ ends of FIP and BIP primers, respectively (Eurofins Genomics; Table 2). LAMP primer concentration and reaction conditions are the same as described above. The assay optimisation was performed according to kit manufacturer instructions. However, the concentration of the analyte added to the LF hybridisation solution was adjusted to avoid false positives. During this optimisation, only the positive control gDNA and NTC were used in three technical replicates (Supplementary Fig. 6). Optimal dilution with double distilled water of the tagged LAMP amplicons was established at 1:9. Briefly, the LF-LAMP assay was carried out as follows: each dipstick was loaded with each of the single test concentrations (Table 3) of the LAMP amplicons on its sample loading strip and dipped vertically into the wells of a 96-wells plate already filled with 100 μL kit buffer solution (Supplementary Fig. 6) and incubated at room temperature. The results were recorded at two intervals of 5 and 10 min. Positive tests showed both stained Control and Test bands. Following the establishment of assay conditions, the assay was tested further on three random field samples.

**Table 3:** Optimisation for the tagged LAMP amplicon concentration for the LF assay.

LF Dipstick ID	Loading Sample and Kit Buffer Volumes
RX1a	1 μL amplicon + 100 μL buffer
RX1b	0.25 μL amplicon + 100 μL buffer
RX1c	(1 μL amplicon + 9 μL molecular grade water) + 100 μL buffer

NTCa	1 μL amplicon + 100 μL buffer
NTCb	0.25 μL amplicon + 100 μL buffer
NTCb	(1 μL amplicon + 9 μL molecular grade water) + 100 μL buffer

### Centrifuge-free/crude DNA extractions

The low *g* centrifuge machine used here was the FVL-2400 Combi-Spin Centrifuge/Vortex (BioSan, Latvia) providing a maximum of 700 ×*g* centrifugation and a medium strength vortexing function. All the experiments were performed in three technical replicates. The DNA quantifications were performed with a Synergy HTX Multi-Mode Microplate Reader (BioTek) using Gen5 Software (version 3.03, BioTek).

*Magnetic beads based method:* This was conducted using the commercially available magnetic beads based genesig^®^ Easy DNA/RNA Extraction Kit (Primerdesign Ltd, UK). The samples used here were the PEK, and separately pooled faecal samples of Farm IV and Farm VI. Both the direct faecal samples and the solution from the Fill-FLOTAC^®^ were used as the starting material, adhering strictly to the amount and the protocols described in the kit manual.

**Fig. 1:**
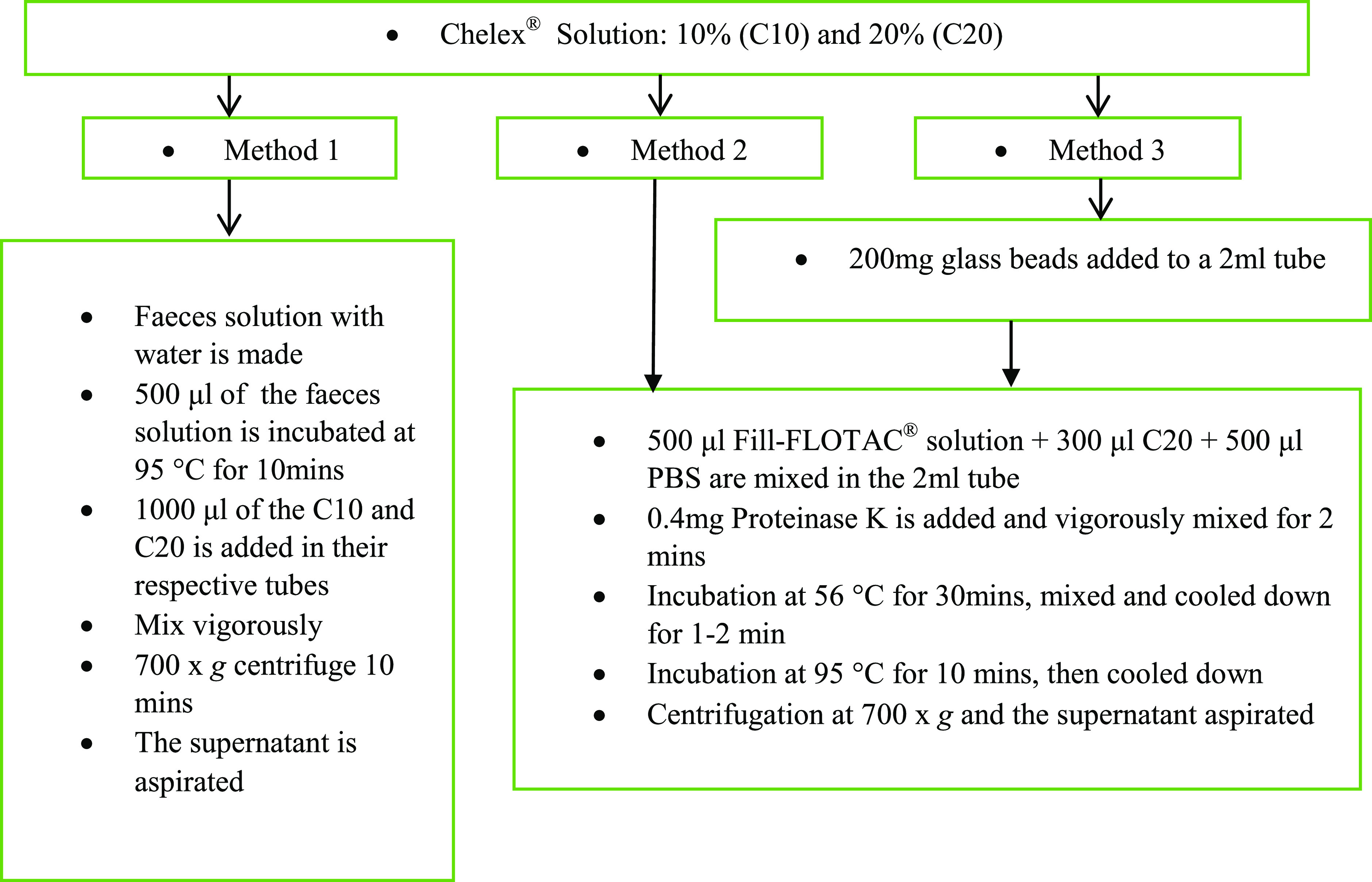
Steps for the crude DNA extractions trials based on Chelex^®^ reagent.

*Chelex*^®^ *Reagent based methods:* Three different trials were conducted using Chelex^®^ 100 (BioRad) solution-based crude DNA extractions from faecal samples and nematodes following a few protocols and studies [2, 18] which were proven effective for other species and at the field level. The modified protocols and the sample amounts utilised are briefly described in Figure 1. The Proteinase K and the glass beads used were from the E.Z.N.A^®^ Stool DNA Kit (Omega Bio-Tek, Inc.).

#### Bead beating crude DNA extraction

Extraction was performed as described in [33] with a slight modification to integrate the Fill-FLOTAC^®^ device keeping in view the POC settings. Briefly, a Fill-FLOTAC^®^ solution was prepared using the Farm I sample in saturated salt solution, as per protocols described above. All of the contents were sieved through a 250 μm filter sieve (Thermo Scientific) into a beaker. To concentrate and harvest the egg pellets as much as possible, the following steps were done: the filtered Fill-FLOTAC^®^ solution was replicated in multiple 2 mL tubes (12 technical replicates as the device accommodates 12 slots for centrifugation) and centrifuged at maximum (~700 ×*g*) using a FVL-2400 Combi-Spin Centrifuge/Vortex (BioSan, Latvia) for 5 min; 250 μL each of the supernatant were transferred to new 2 mL tubes; distilled water was added to each of them to make the volume 2 mL and centrifuged at maximum for 10 min; the pellets at the bottom were carefully collected, pooled into three technical replicates to maximise the yield and washed. A small portion from one of the replicates was observed under a microscope to verify egg presence. The egg pellets were stored with 500 μL distilled water in 2 mL tubes. Finally, for the bead beating lysis, 200 mg of glass beads (E.Z.N.A^®^ Stool DNA Kit, Omega Bio-Tek, Inc.) were added to each 2 mL tube containing the egg pellets and vortexed for 5 min. Additionally, bead beating lysis was also carried out directly using the Fill-FLOTAC^®^ faecal homogenate solution but without the egg concentration steps described above. One μL of lysate from each sample/replicate was diluted 1:5 with double-distilled H_2_O and used as the template per LAMP reaction.

### Single-species specific PCR

*Haemonchus contortus* species-specific ITS2 PCR was set up using a GoTaq Flexi Polymerase PCR kit (Promega), according to the manufacturer’s instructions. The *H. contortus* specific ITS2 primer set (Eurofins Genomics) used herein was initially described by Redman et al. (2008) [43] (Table 2). ITS2 PCR was set up in 12.5 μL reaction volume as follows: 2.5 μL 5× GoTaq Green Buffer, 1.25 μL MgCl_2_ 25 mM, 0.25 μL dNTPs 10 mM each (VWR International), 0.25 μL (100 pmol) each of Forward and Reverse primers, 0.06 μL GoTaq Flexi polymerase 5U/μL (Promega), 6.94 μL of molecular grade water (AccuGene) and 1 μL of DNA template. The thermal profile of the PCR was as follows: denaturation at 94 °C for 2 min; 35 cycles each of 94 °C 30 s, the annealing temperature of 50 °C for 30 s and 72 °C extension for 30 s, with a final extension step of 72 °C 10 min. Visualisation of results was carried out using ChemiDoc XRS+ System and Image Lab (BioRad) after gel electrophoresis in 2% agarose gel. The amplicon size was 320 bp as per [43]. Farms I–X, Farm DF-GF and gDNA of the positive control PEK were utilised in this PCR and the subsequent LAMP assay.

## Results

### Colourimetric LAMP assay

**Fig. 2:**
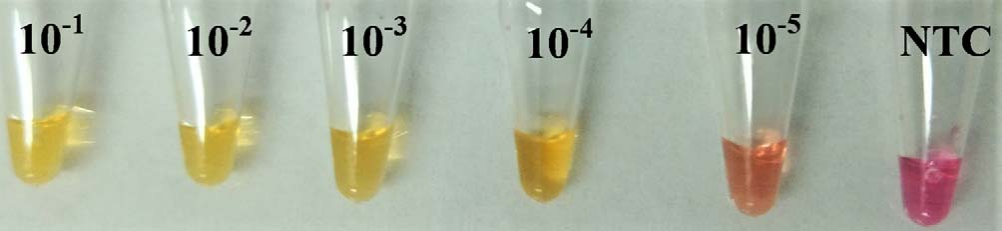
Sensitivity of the colourimetric LAMP assay as expressed through a 10-fold serial dilution of positive control gDNA of *H. contortus*. NTC: Non-template control.

Initial optimisation of the assay was necessary to determine the optimal temperature for the reaction using the WarmStart^®^ Colorimetric LAMP 2× Master Mix. This was determined to be 62 °C using confirmed *H. contortus* genomic DNA. The assay was initially run for a maximum of 45 min although subsequently a 30 min reaction time was found to be adequate. Positive results showed a change from pink/purple to yellow/orange as given in the kit protocols. All the controls used during the experiment did not show any false results, thus confirming the specificity of the primer sets (Supplementary Fig. 1). The detection range of the assay was found to be 2.5 × 10^−1^ ng/μL to 2.5 × 10^−5^ ng/μL based on the 10-fold serially diluted template LAMP assays (Fig. 2). A positive result was also successfully detected in sheep faecal samples from Farm I and Farm III. gDNA extracted from the suspected *H. contortus* adult worms, obtained from the abomasum at post mortem from a farmed deer (Farm DF), showed positive results while the DNA samples of a few unidentified worms collected from the large intestines of the same deer were shown to be negative by the assay. Another two faecal samples from goats (Farm GF) were also confirmed positive for *H. contortus* (Supplementary Fig. 2). However, pooled faecal samples (Farm II) of individual sheep with no trichostrongyle egg counts observed during FEC yielded negative LAMP results (Supplementary Fig. 3), although subsequent LAMP trials of pooled samples from the same farm inclusive of all individual samples were found to give a positive result.

We followed up on these results by expanding our sample size and also including samples from individual sheep rather than pooled samples to verify the diagnostic sensitivity for individuals as well as flocks. Faecal samples from Farm VI were already found to have a very low average egg count (EPG = 5.96) and no detectable trichostrongyle eggs by microscopic analysis (Toth et al., unpublished). Our LAMP analysis of randomly selected individuals samples of Farm VI was found to have negative results (Supplementary Fig. 3: A). Farm IV faecal samples have very high trichostrongyle egg counts with some individual samples suspected of having *H. contortus* eggs. LAMP assay using these individual samples as well as pooled samples detected positive results (Supplementary Fig. 3: B1–5, C1–10). Similarly, pooled and individual samples were examined from Farm VIII and also tested positive (Supplementary Fig. 3: D1–5).

### LAMP validation by PCR

**Fig. 3:**
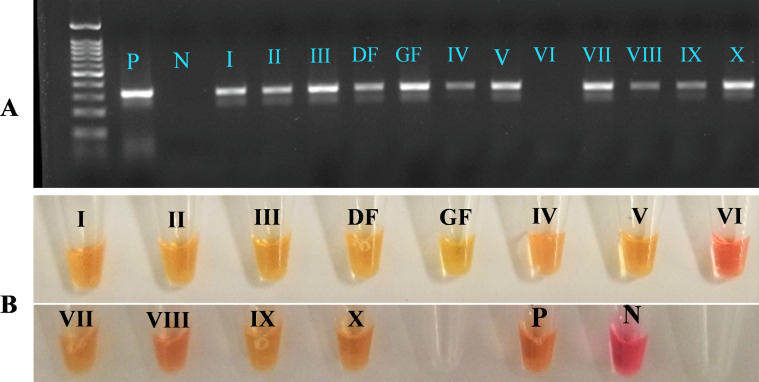
Validation of the colourimetric LAMP assay by the ITS2 PCR. A: Gel-electrophoresis image of PCR amplicons. Ladder used: 100 bp ladder. B: Corresponding LAMP results. P: Positive control. N: Non-template control. I-X: Farm I-X. DF: Farm DF. GF: Farm GF. Yellow/Orange = Positive/Weak Positive, Pink/Purple = Negative.

Following the predicted detection of *H. contortus* in the above-mentioned farms, ITS2 Speciation PCR was carried out to validate the LAMP assay. Farms I–V, VII–X, DF (suspected *H. contortus* adult) and GF were all shown to have positive results while Farm VI showed negative results in both the PCR and LAMP. None of the controls used showed false results. This showed that there was 100% agreement between the two assays (Fig. 3).

### DNA extraction methods

The DNA measurements are given in Table 4. No positive LAMP results were obtained from the Chelex^®^ reagent-based methods. Also, the PEK samples for Chelex^®^ Method 1 could not yield any reading as the analyte was not clear enough for the absorbance reading. The genesig^®^ Easy DNA/RNA Extraction Kit (Primerdesign Ltd, UK) which utilises a magnetic bead extraction protocol gave inconsistent LAMP results. One replicate of the positive sample PEK showed a negative result. Yet, both replicates from Farm IV showed positive results while that of Farm VI showed negative results, as expected (Supplementary Fig. 4). For the bead-beating technique, the supernatant from the direct Fill-FLOTAC^®^ solution did not yield any positive results by LAMP (Supplementary Fig. 5: A). However, the protocol was slightly modified by using the benchtop low *g* centrifuge. This led to an improvement in the detection of *H. contortus* DNA from the sample used as two out of three technical replicates gave positive LAMP results (Supplementary Fig. 5: B).

**Table 4:** DNA measurements for the supplementary DNA extraction methods.

Method Name	Sample ID	Average contamination Ratio (260/230)	Average purity ratio (260/280)	Average yield (ng/mL)
Magnetic Beads Kit	PEK ^DT^	0.76	1.08	33.67
	PEK ^F^	0.72	1.12	115
	Farm IV ^DT^	0.66	1.06	40.65
	Farm IV^F^	0.68	1.09	24.82
	Farm VI^DT^	0.71	1.08	27.42
	Farm VI^F^	0.61	1.05	18.32
Chelex^®^ Method 1	PEK ^DT^ C10	NA	NA	NA
	PEK ^DT^ C20	NA	NA	NA
	Farm IV^DT^ C10	0.65	1.09	181.87
	Farm IV^DT^ C20	0.65	1.09	207.32
	Farm VI^DT^ C10	0.67	1.14	99.47
	Farm VI^DT^ C20	0.67	1.13	106.27
Chelex^®^ Method 2	PEK ^F^	0.59	0.95	34.45
	Farm IV^F^	0.59	0.95	33.5
	Farm VIII^F^	0.28	1.47	9.6
Chelex^®^ Method 3	PEK ^F^	0.51	1.04	164.35
	Farm IV^F^	0.50	1.04	136.45
	Farm VI^F^	0.42	1.02	81.15
Bead-beating Tube Method	Farm I^F^	0.34	1.05	63
	Farm I ^Centrifuged^	0.58	1.09	14.25

F: Fill-FLOTAC Solution; DT: Direct Faecal Sample. PEK: Positive control sample.

### Lateral flow assay

**Fig. 4:**
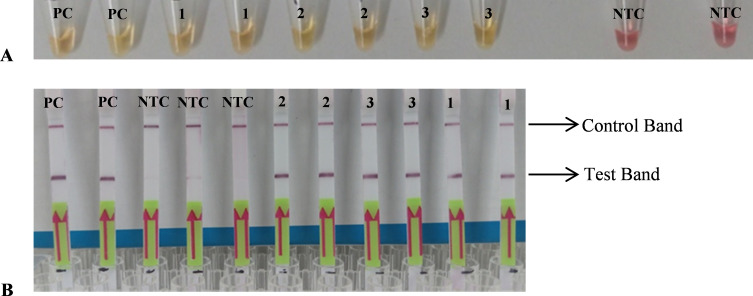
LF-LAMP assay. Optimised assay tested against three random farm samples. A: LAMP assay using tagged primers. Yellow/Orange = Positive/Weak Positive, Pink/Purple = Negative. B: LF-LAMP assay using the tagged LAMP amplicons. Positive detection of *H. contortus* presents as a band in both Control and Test lines. Negative detection presents as a band in only the Control line. Failed assay presents as no bands on LF strip. 1: Farm V. 2: Farm VIII. 3: Farm X. NTC: Non-template control. PC: Positive control gDNA of *H. contortus.*

Our initial optimisation was carried out using our *H. contortus* positive control gDNA (from both PEK and PCB) and non-template controls. Results showed that the LF was capable of detecting these two positive control gDNA samples (Supplementary Fig. 6: 1–3) and with no false-positive bands visible for the non-template control (Supplementary Fig. 6: 4–6). Finally, the amplicons obtained using samples from Farm V, Farm VIII and Farm IX were used to test the accuracy of the LF (Fig. 4). We found 100% agreement between the colourimetric LAMP (as already confirmed by the ITS2 PCR) and the LF-LAMP assays. There was no appreciable difference seen in the strength of the positives between the field samples and the *H. contortus* positive control gDNA (Fig. 4). Thus, the LF assay was determined to be optimised for the detection of *H. contortus* DNA using field samples.

## Discussion

In this study, we built on the previous work by Melville et al. (2014) [33] to develop a proof of concept study for a POC confirmatory diagnostic test that can qualitatively detect *H. contortus*. Besides a few centrifuge-free methods for crude DNA extraction, we also tested one using the glass bead-beating method already done in the same original study mentioned above. The primer set in use had already been tested for *H. contortus* specificity against a collection of closely related nematode species [33]. We expanded the scope of the assay from the original study by utilising a commercially available colourimetric kit for a naked eye detection system. The sensitivity of our assay was also found to be close to that of the original study, based on the result of the serial dilution (Fig. 2). The slight decrease in the sensitivity of our assay from that of the original study might be due to the difference in the detection system. While the original study used the UV illumination technique, our assay merely relies on naked-eye detection of colour change. However, this colourimetric detection offers the advantage of minimal equipment usage for a POC diagnosis system.

Initial optimisation of the colourimetric LAMP assay was achieved using positive control gDNA samples from Slovakia as well as in Hungary (PEK and PCB, respectively). Once reaction conditions were established, we further validated the assay on several field samples obtained from various farms in Eastern and South-Eastern Hungary. The purpose of using these field samples was two-fold. In the first instance, we attempted to further validate the primers published in [33] and to demonstrate that they were effective in a geographically and genetically distinct population of *H. contortus* from that tested in the original study. This was necessary as *H. contortus* displays a great degree of both inter- and intra-isolate diversity [14, 27, 47]. We found that the results of our LAMP assay were in 100% agreement with those of the established ITS2 speciation PCR (Fig. 3), which served as confirmation of the presence of *H. contortus* within those samples. It is also worth mentioning that Farm I had a history of clinical haemonchosis and had also been diagnosed for it by an independent veterinary laboratory. Subsequent analysis of the faecal samples by the Peanut Agglutination (PNA) fluorescence microscopy for Farm I already detected *H. contortus* eggs (Supplementary Fig. 7). Also, Farm VI had a record of low EPG and our LAMP assay for Farm VI also gave negative results (Supplementary Fig. 3: A). Subsequent tests of the optimised LAMP on DNA from individuals as well as pooled samples gave no false results. It should be noted here that Farm II had a significant number of eggs when counted by microscopy but only a few samples had suspected trichostrongyle eggs. Thus, two batches of pooled samples were prepared: one excluding the suspected trichostrongyle egg samples and another inclusive of all. LAMP assay showed negative and positive results, respectively for these two pooled samples. All the above evidence affirms that the assay can qualitatively predict the presence of *H. contortus.* If this qualitative detection is incorporated with FEC figures obtained on the spot, for instance by using Mini-FLOTAC^®^ with a battery-operated portable microscope, it could aid in giving clinically relevant information to the farmers. The idea of minimal equipment usage was also the reason behind the adoption and use of the small-sized mini heat-blocks (VWR^®^ Advanced Mini Dry Block Heaters) throughout all LAMP runs following the initial optimisation. This yielded positive results and was indistinguishable from the use of a commercial thermocycler. Our minimal equipment set up has thus been designed with the POC in mind, with each piece of equipment requiring low power input which could easily be supplied by a portable generator.

We then sought to determine if we could integrate existing centrifuge-free DNA extraction protocols by using Chelex^®^ reagent and magnetic bead-based DNA separation as well as the glass-beads beating method using a portable but low-power centrifuge. All these various methods mentioned above were done with the aim of reducing the need for sophisticated laboratory equipment at the farm as well as to obtain DNA in as short a time as possible given the POC setting. We considered this an important inclusion in the study as DNA extraction presents the current rate-limiting step, owing to the requirement of high-grade centrifugation equipment in most of the commercial kits. Proteinase K digestion is commonly used to produce crude lysates, or commercial kits using silica membrane-based technology for DNA capture [39] but this methodology is time-consuming and requires long digestion and inactivation times. Although the Chelex^®^ reagent-based DNA extraction was already shown to be effective in crude DNA lysis for other target samples, a concrete result could not be obtained in our case. This might be attributed to the comparative difficulty in breaking the tough egg wall of the parasite in question.

Of the centrifuge-free DNA extraction methods tested, the genesig^®^ Easy DNA/RNA Extraction Kit (Primerdesign Ltd, UK) yielded promising results. Comparing DNA yields (Table 1), it was found that there was a lower yield obtained with magnetic bead extraction, although this is offset by the ease of POC amenability of this kit. Finally, glass bead-beating lysis was also tested, but this gave a positive result in two out of the three replicates for those with egg concentration steps. This inconsistency could be due to inadequate eggs in the pellets obtained and/or the low power vortexing (and centrifugation) ability of the FVL-2400 Combi-Spin Centrifuge/Vortex (BioSan, Latvia) thus resulting in a reduced bead-beating effect to open the eggs. This technique could be improved if a larger starting volume for the pellets was available and also by using a stronger centrifugation/vortexing device with adequate farm site adaptability. The low *g* centrifuge machine mentioned above (maximum 700 ×*g*), although highly amenable to the POC setting, lacked the efficiency to give clear/clean final analytes due to the presence of high amounts of faecal debris. Thus, our recommendation moving forward would be to further test the magnetic-bead based method in a larger study to determine whether results are replicable and diagnostic sensitivity is not overly compromised.

Finally, we made use of a commercially available and ready optimised lateral flow assay kit to optimise and demonstrate a proof of concept LF-LAMP for POC amenable confirmatory diagnostic test for *H. contortus.* To our knowledge at the time of writing, this study constitutes the first demonstration of an LF-LAMP assay for the species-specific colourimetric confirmatory diagnosis of *H. contortus.* The LF-LAMP was tested in field samples (Table 2) previously predicted to be positive for *H. contortus* by LAMP and confirmed by PCR (Fig. 3). We found 100% agreement between the results of our colourimetric LAMP assay and the LF-LAMP assay, indicating the validity of either of the endpoint detection methods for potential future POC confirmatory diagnosis of *H. contortus.* However, our proof of concept LF-LAMP assay is still purely qualitative and thus cannot seek to replace quantitative microscopy and/or other molecular-based methods at present. Nevertheless, significant improvements have been made in recently published studies detailing the use of smartphone-based technologies demonstrating proof of concept point-of-care quantitative diagnosis using a colourimetric LAMP assay [54]. This presents an important avenue for future research towards creating molecular assays that can replace traditional microscopy by delivering both species-specific identification and quantification.

In summary, the proof of concept study outlined here is of value for the potential improvement of farm site species-specific identification of important GIN parasites. Simple visual-based endpoint detection removes the need for laboratory-based and equipment heavy methodologies such as gel electrophoresis visualisation or fluorescence detection. Colourimetric detection is more immediately amenable to the detection of a single organism in a one-tube assay, and thus could at this stage be used for on-site confirmatory diagnosis of a single parasite of interest following an established FEC examination/detection in a mixed infection scenario. As veterinary GIN infections are very rarely single species [32, 45], it is important that any molecular test should be multiplexable. There are already commercially available universal LF paper strip kits that are claimed to successfully detect at least two separate analytes. We envision that the concept presented here would provide the foundation for a multi-species colourimetric single-tube LAMP assay. For instance, this LAMP assay could be multiplexed using any proven species-specific primers (and subsequently tagged with distinct epitopes per species) of GIN parasites of interest. This initial colourimetric LAMP assay could first be used for a simple yes/no answer as to the presence of GIN infection. Subsequently, a multiplexed LF assay could then make species-specific identification following similar methodology demonstrated in other pathogens and infections [16]. However, significant further validation would be required before this could be adapted into a commercial assay suitable for farm site diagnosis. Nevertheless, by building on existing protocols and established techniques, suitable on-site FEC techniques such as Mini-FLOTAC^®^ and existing commercial assays, we can move closer to developing a farm site molecular diagnostic speciation with clinically relevant information.

## Conflict of interest

The authors report that they have no conflict of interest.

## Supplementary Materials

The Supplementary materials of this article are available at https://www.parasite-journal.org/10.1051/parasite/2021078/olm. *Supplementary Figure 1*: Optimisation of Colourimetric LAMP Assay with positive control gDNA of *H. contortus*. 1: 60 °C. 2: 61 °C. 3: 62 °C. 4: Non-template control. 30–45 min reaction time.
*Supplementary Figure 2*: Colourimetric LAMP assay validation on suspected *H. contortus* field samples. 1: Positive Control (PEK). 2: Farm I. 3: Farm III. 4, 9 & 14: Positive control *H. contortus* gDNA (PEB). 5 & 6: Suspected adult *H. contortus* worms from deer (Farm DF). 7 & 8: Unidentified adult worms from deer large intestine (Farm DF). 10: Farm II. 12 & 13: Goat faeces sample **(**Farm GF). Yellow/Orange = Positive/Weak Positive, Pink/Purple = Negative.
*Supplementary Figure 3*: Colourimetric LAMP assay validation on suspected *H. contortus* field samples using the individual as well as pooled faecal samples. A: Farm VI. 1-5: Individual sheep samples (random). 6: Positive control *H. contortus* gDNA. 7: No Template Control. B: Farm IV. 1-5: Individual sheep samples (random). 6: Positive control *H. contortus* gDNA. 7: Non-template control. C: Farm IV. 1-10: Pooled samples. 11: Positive control *H. contortus* gDNA. 12: No Template Control. D: Farm VIII. 1-3: Pooled samples. 4 & 5: Individual sheep samples (random). 6: Positive control *H. contortus* gDNA. 7: No Template Control. Yellow/Orange = Positive/Weak Positive, Pink/Purple = Negative.
*Supplementary Figure 4*: LAMP Assay with *H. contortus* egg DNA extracted by genesig^®^ DNA/RNA Easy Magnetic Bead Extraction (Primerdesign Ltd, UK). 1 & 2: Positive control (PEK). 4: Farm IV. 5 & 6: Farm VI. 7: Non-template Control. 8: Positive control *H. contortus* gDNA. Yellow/Orange = Positive/Weak Positive, Pink/Purple = Negative.
*Supplementary Figure 5*: Bead-beating lysis and DNA extraction. Farm I samples. A: Direct Fill-FLOTAC^®^ solution. B: Fill-FLOTAC^®^ solution after low *g* centrifugation egg harvesting steps. PC: Positive control. NTC: Non-template control.
*Supplementary Figure 6*: LF-LAMP optimisation results using diluted tagged amplicons. 1-3: Positive control *H. contortus* gDNA. 4-6: Non-template control. Positive detection of *H. contortus* presents as a band in both control and test lines. Negative detection presents as a band in only the control line. Failed assay presents as no bands on LF strip.
*Supplementary Figure 7*: Peanut Agglutination (PNA) fluorescence microscopy result of Farm I (Khangembam et al, unpublished) performed as per Jurasek et al (2010). 1 & 2: Different fields of view of *H. contortus* eggs seen as bright-green stained eggs. 10x magnification.
