# Supplementary figures and images for: Point of care colourimetric and lateral flow LAMP assay for the detection of Haemonchus contortus in ruminant faecal samples
Source: Parasite. 2021 Dec 15;28:82. doi: 10.1051/parasite/2021078 (PMC8672678; doi:10.1051/parasite/2021078)

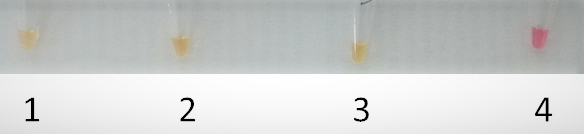

Supplement: Supplementary file 1 — Supplementary Figure 1: Optimisation of Colourimetric LAMP Assay with positive control gDNA of H. contortus. 1: 60 °C. 2: 61 °C. 3: 62 °C. 4: Non-template control. 30–45 min reaction time. Supplementary Figure 2: Colourimetric LAMP assay validation on suspected H. contortus field samples. 1: Positive Control (PEK). 2: Farm I. 3: Farm III. 4, 9 & 14: Positive control H. contortus gDNA (PEB). 5 & 6: Suspected adult H. contortus worms from deer (Farm DF). 7 & 8: Unidentified adult worms from deer large intestine (Farm DF). 10: Farm II. 12 & 13: Goat faeces sample (Farm GF). Yellow/Orange = Positive/Weak Positive, Pink/Purple = Negative. Supplementary Figure 3: Colourimetric LAMP assay validation on suspected H. contortus field samples using the individual as well as pooled faecal samples. A: Farm VI. 1-5: Individual sheep samples (random). 6: Positive control H. contortus gDNA. 7: No Template Control. B: Farm IV. 1-5: Individual sheep samples (random). 6: Positive control H. contortus gDNA. 7: Non-template control. C: Farm IV. 1-10: Pooled samples. 11: Positive control H. contortus gDNA. 12: No Template Control. D: Farm VIII. 1-3: Pooled samples. 4 & 5: Individual sheep samples (random). 6: Positive control H. contortus gDNA. 7: No Template Control. Yellow/Orange = Positive/Weak Positive, Pink/Purple = Negative. Supplementary Figure 4: LAMP Assay with H. contortus egg DNA extracted by genesig® DNA/RNA Easy Magnetic Bead Extraction (Primerdesign Ltd, UK). 1 & 2: Positive control (PEK). 4: Farm IV. 5 & 6: Farm VI. 7: Non-template Control. 8: Positive control H. contortus gDNA. Yellow/Orange = Positive/Weak Positive, Pink/Purple = Negative. Supplementary Figure 5: Bead-beating lysis and DNA extraction. Farm I samples. A: Direct Fill-FLOTAC® solution. B: Fill-FLOTAC® solution after low g centrifugation egg harvesting steps. PC: Positive control. NTC: Non-template control. Supplementary Figure 6: LF-LAMP optimisation results using diluted tagged amplicons. 1-3: Positive con [file parasite-28-82-s1.zip › parasite210006-1-olm/Supplementary Figure 1.jpg]

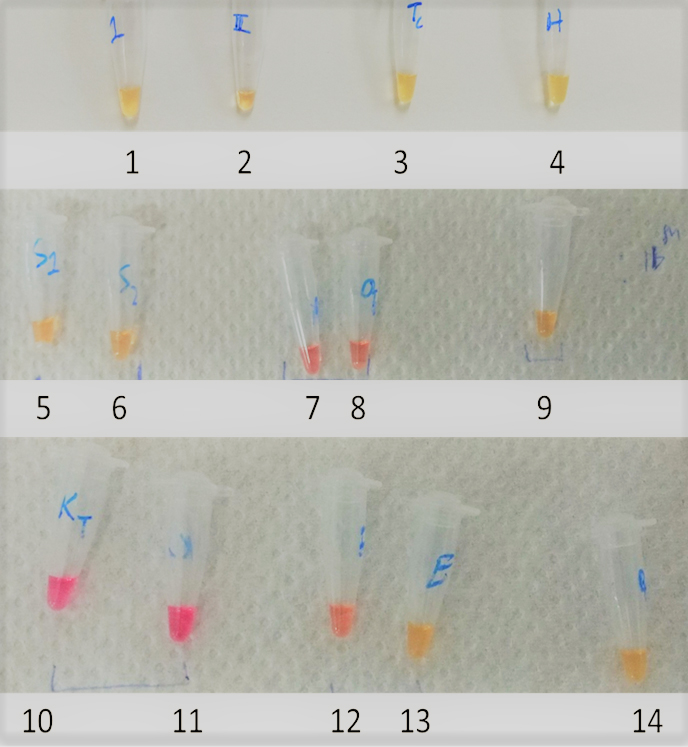

Supplement: Supplementary file 1 — Supplementary Figure 1: Optimisation of Colourimetric LAMP Assay with positive control gDNA of H. contortus. 1: 60 °C. 2: 61 °C. 3: 62 °C. 4: Non-template control. 30–45 min reaction time. Supplementary Figure 2: Colourimetric LAMP assay validation on suspected H. contortus field samples. 1: Positive Control (PEK). 2: Farm I. 3: Farm III. 4, 9 & 14: Positive control H. contortus gDNA (PEB). 5 & 6: Suspected adult H. contortus worms from deer (Farm DF). 7 & 8: Unidentified adult worms from deer large intestine (Farm DF). 10: Farm II. 12 & 13: Goat faeces sample (Farm GF). Yellow/Orange = Positive/Weak Positive, Pink/Purple = Negative. Supplementary Figure 3: Colourimetric LAMP assay validation on suspected H. contortus field samples using the individual as well as pooled faecal samples. A: Farm VI. 1-5: Individual sheep samples (random). 6: Positive control H. contortus gDNA. 7: No Template Control. B: Farm IV. 1-5: Individual sheep samples (random). 6: Positive control H. contortus gDNA. 7: Non-template control. C: Farm IV. 1-10: Pooled samples. 11: Positive control H. contortus gDNA. 12: No Template Control. D: Farm VIII. 1-3: Pooled samples. 4 & 5: Individual sheep samples (random). 6: Positive control H. contortus gDNA. 7: No Template Control. Yellow/Orange = Positive/Weak Positive, Pink/Purple = Negative. Supplementary Figure 4: LAMP Assay with H. contortus egg DNA extracted by genesig® DNA/RNA Easy Magnetic Bead Extraction (Primerdesign Ltd, UK). 1 & 2: Positive control (PEK). 4: Farm IV. 5 & 6: Farm VI. 7: Non-template Control. 8: Positive control H. contortus gDNA. Yellow/Orange = Positive/Weak Positive, Pink/Purple = Negative. Supplementary Figure 5: Bead-beating lysis and DNA extraction. Farm I samples. A: Direct Fill-FLOTAC® solution. B: Fill-FLOTAC® solution after low g centrifugation egg harvesting steps. PC: Positive control. NTC: Non-template control. Supplementary Figure 6: LF-LAMP optimisation results using diluted tagged amplicons. 1-3: Positive con [file parasite-28-82-s1.zip › parasite210006-1-olm/Supplementary Figure 2.jpg]

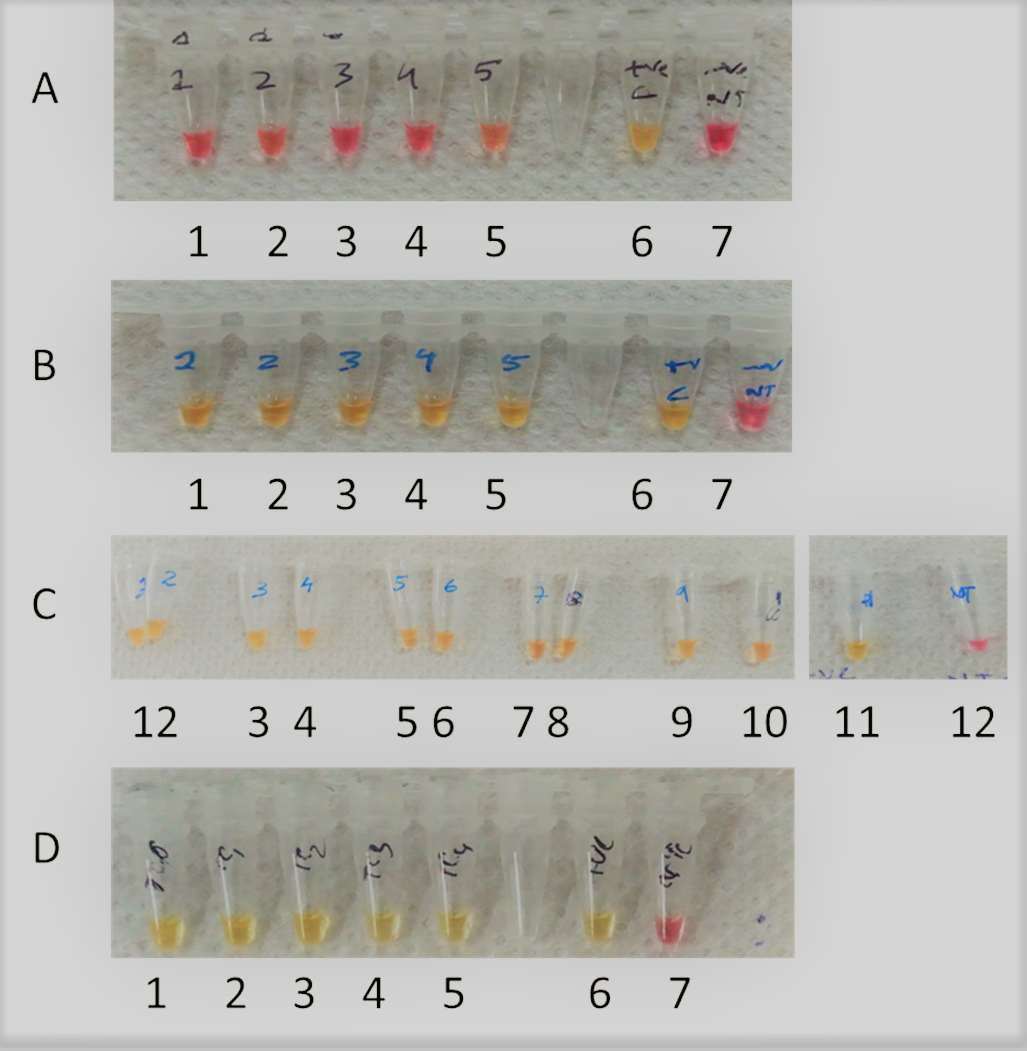

Supplement: Supplementary file 1 — Supplementary Figure 1: Optimisation of Colourimetric LAMP Assay with positive control gDNA of H. contortus. 1: 60 °C. 2: 61 °C. 3: 62 °C. 4: Non-template control. 30–45 min reaction time. Supplementary Figure 2: Colourimetric LAMP assay validation on suspected H. contortus field samples. 1: Positive Control (PEK). 2: Farm I. 3: Farm III. 4, 9 & 14: Positive control H. contortus gDNA (PEB). 5 & 6: Suspected adult H. contortus worms from deer (Farm DF). 7 & 8: Unidentified adult worms from deer large intestine (Farm DF). 10: Farm II. 12 & 13: Goat faeces sample (Farm GF). Yellow/Orange = Positive/Weak Positive, Pink/Purple = Negative. Supplementary Figure 3: Colourimetric LAMP assay validation on suspected H. contortus field samples using the individual as well as pooled faecal samples. A: Farm VI. 1-5: Individual sheep samples (random). 6: Positive control H. contortus gDNA. 7: No Template Control. B: Farm IV. 1-5: Individual sheep samples (random). 6: Positive control H. contortus gDNA. 7: Non-template control. C: Farm IV. 1-10: Pooled samples. 11: Positive control H. contortus gDNA. 12: No Template Control. D: Farm VIII. 1-3: Pooled samples. 4 & 5: Individual sheep samples (random). 6: Positive control H. contortus gDNA. 7: No Template Control. Yellow/Orange = Positive/Weak Positive, Pink/Purple = Negative. Supplementary Figure 4: LAMP Assay with H. contortus egg DNA extracted by genesig® DNA/RNA Easy Magnetic Bead Extraction (Primerdesign Ltd, UK). 1 & 2: Positive control (PEK). 4: Farm IV. 5 & 6: Farm VI. 7: Non-template Control. 8: Positive control H. contortus gDNA. Yellow/Orange = Positive/Weak Positive, Pink/Purple = Negative. Supplementary Figure 5: Bead-beating lysis and DNA extraction. Farm I samples. A: Direct Fill-FLOTAC® solution. B: Fill-FLOTAC® solution after low g centrifugation egg harvesting steps. PC: Positive control. NTC: Non-template control. Supplementary Figure 6: LF-LAMP optimisation results using diluted tagged amplicons. 1-3: Positive con [file parasite-28-82-s1.zip › parasite210006-1-olm/Supplementary Figure 3.jpg]

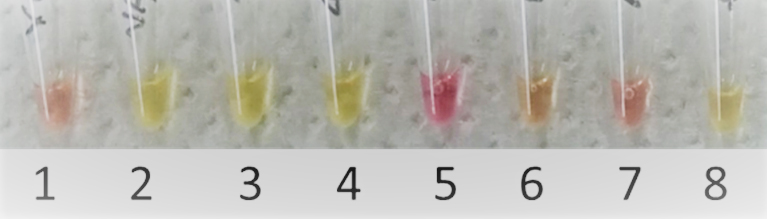

Supplement: Supplementary file 1 — Supplementary Figure 1: Optimisation of Colourimetric LAMP Assay with positive control gDNA of H. contortus. 1: 60 °C. 2: 61 °C. 3: 62 °C. 4: Non-template control. 30–45 min reaction time. Supplementary Figure 2: Colourimetric LAMP assay validation on suspected H. contortus field samples. 1: Positive Control (PEK). 2: Farm I. 3: Farm III. 4, 9 & 14: Positive control H. contortus gDNA (PEB). 5 & 6: Suspected adult H. contortus worms from deer (Farm DF). 7 & 8: Unidentified adult worms from deer large intestine (Farm DF). 10: Farm II. 12 & 13: Goat faeces sample (Farm GF). Yellow/Orange = Positive/Weak Positive, Pink/Purple = Negative. Supplementary Figure 3: Colourimetric LAMP assay validation on suspected H. contortus field samples using the individual as well as pooled faecal samples. A: Farm VI. 1-5: Individual sheep samples (random). 6: Positive control H. contortus gDNA. 7: No Template Control. B: Farm IV. 1-5: Individual sheep samples (random). 6: Positive control H. contortus gDNA. 7: Non-template control. C: Farm IV. 1-10: Pooled samples. 11: Positive control H. contortus gDNA. 12: No Template Control. D: Farm VIII. 1-3: Pooled samples. 4 & 5: Individual sheep samples (random). 6: Positive control H. contortus gDNA. 7: No Template Control. Yellow/Orange = Positive/Weak Positive, Pink/Purple = Negative. Supplementary Figure 4: LAMP Assay with H. contortus egg DNA extracted by genesig® DNA/RNA Easy Magnetic Bead Extraction (Primerdesign Ltd, UK). 1 & 2: Positive control (PEK). 4: Farm IV. 5 & 6: Farm VI. 7: Non-template Control. 8: Positive control H. contortus gDNA. Yellow/Orange = Positive/Weak Positive, Pink/Purple = Negative. Supplementary Figure 5: Bead-beating lysis and DNA extraction. Farm I samples. A: Direct Fill-FLOTAC® solution. B: Fill-FLOTAC® solution after low g centrifugation egg harvesting steps. PC: Positive control. NTC: Non-template control. Supplementary Figure 6: LF-LAMP optimisation results using diluted tagged amplicons. 1-3: Positive con [file parasite-28-82-s1.zip › parasite210006-1-olm/Supplementary Figure 4.jpg]

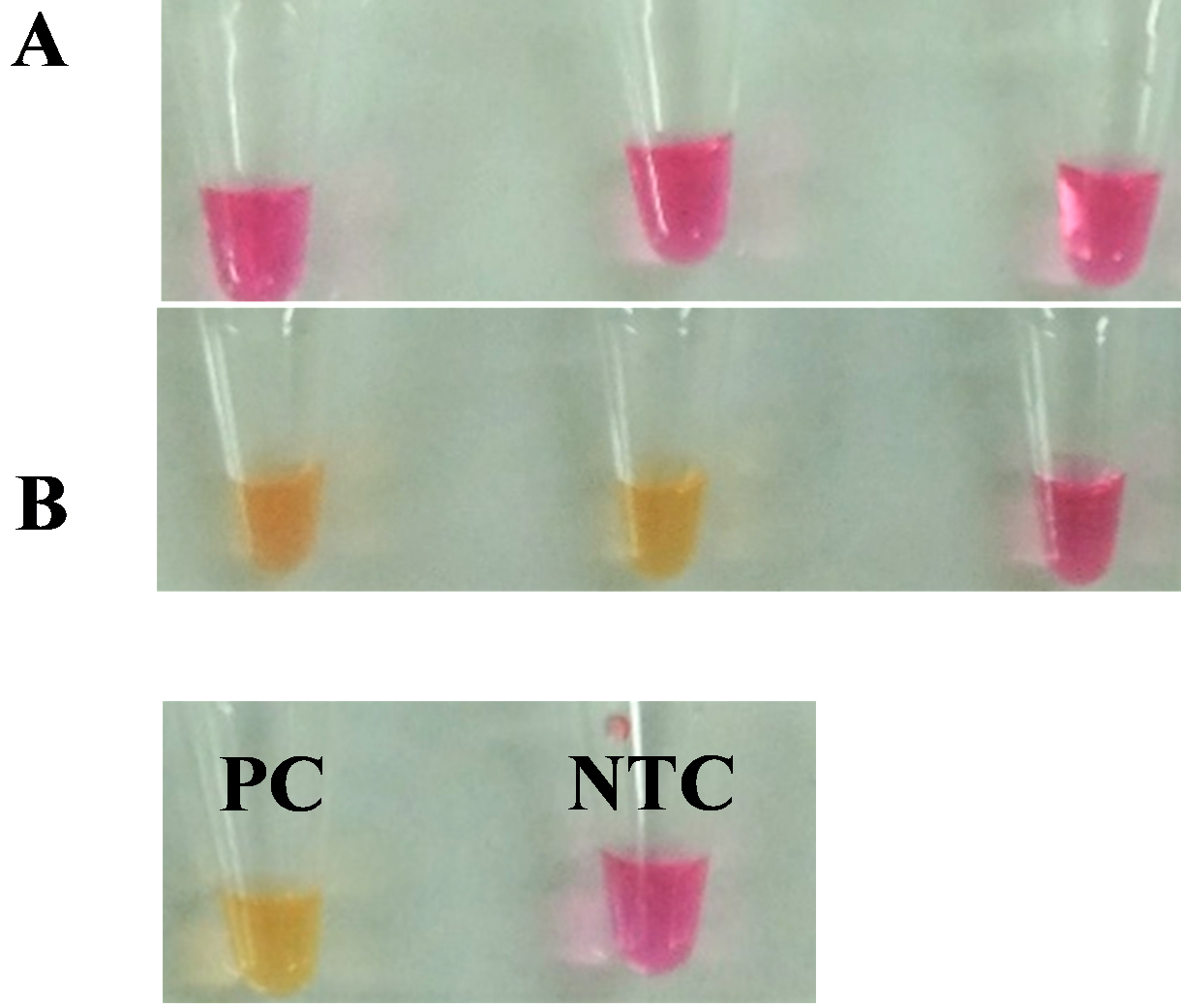

Supplement: Supplementary file 1 — Supplementary Figure 1: Optimisation of Colourimetric LAMP Assay with positive control gDNA of H. contortus. 1: 60 °C. 2: 61 °C. 3: 62 °C. 4: Non-template control. 30–45 min reaction time. Supplementary Figure 2: Colourimetric LAMP assay validation on suspected H. contortus field samples. 1: Positive Control (PEK). 2: Farm I. 3: Farm III. 4, 9 & 14: Positive control H. contortus gDNA (PEB). 5 & 6: Suspected adult H. contortus worms from deer (Farm DF). 7 & 8: Unidentified adult worms from deer large intestine (Farm DF). 10: Farm II. 12 & 13: Goat faeces sample (Farm GF). Yellow/Orange = Positive/Weak Positive, Pink/Purple = Negative. Supplementary Figure 3: Colourimetric LAMP assay validation on suspected H. contortus field samples using the individual as well as pooled faecal samples. A: Farm VI. 1-5: Individual sheep samples (random). 6: Positive control H. contortus gDNA. 7: No Template Control. B: Farm IV. 1-5: Individual sheep samples (random). 6: Positive control H. contortus gDNA. 7: Non-template control. C: Farm IV. 1-10: Pooled samples. 11: Positive control H. contortus gDNA. 12: No Template Control. D: Farm VIII. 1-3: Pooled samples. 4 & 5: Individual sheep samples (random). 6: Positive control H. contortus gDNA. 7: No Template Control. Yellow/Orange = Positive/Weak Positive, Pink/Purple = Negative. Supplementary Figure 4: LAMP Assay with H. contortus egg DNA extracted by genesig® DNA/RNA Easy Magnetic Bead Extraction (Primerdesign Ltd, UK). 1 & 2: Positive control (PEK). 4: Farm IV. 5 & 6: Farm VI. 7: Non-template Control. 8: Positive control H. contortus gDNA. Yellow/Orange = Positive/Weak Positive, Pink/Purple = Negative. Supplementary Figure 5: Bead-beating lysis and DNA extraction. Farm I samples. A: Direct Fill-FLOTAC® solution. B: Fill-FLOTAC® solution after low g centrifugation egg harvesting steps. PC: Positive control. NTC: Non-template control. Supplementary Figure 6: LF-LAMP optimisation results using diluted tagged amplicons. 1-3: Positive con [file parasite-28-82-s1.zip › parasite210006-1-olm/Supplementary Figure 5.jpg]

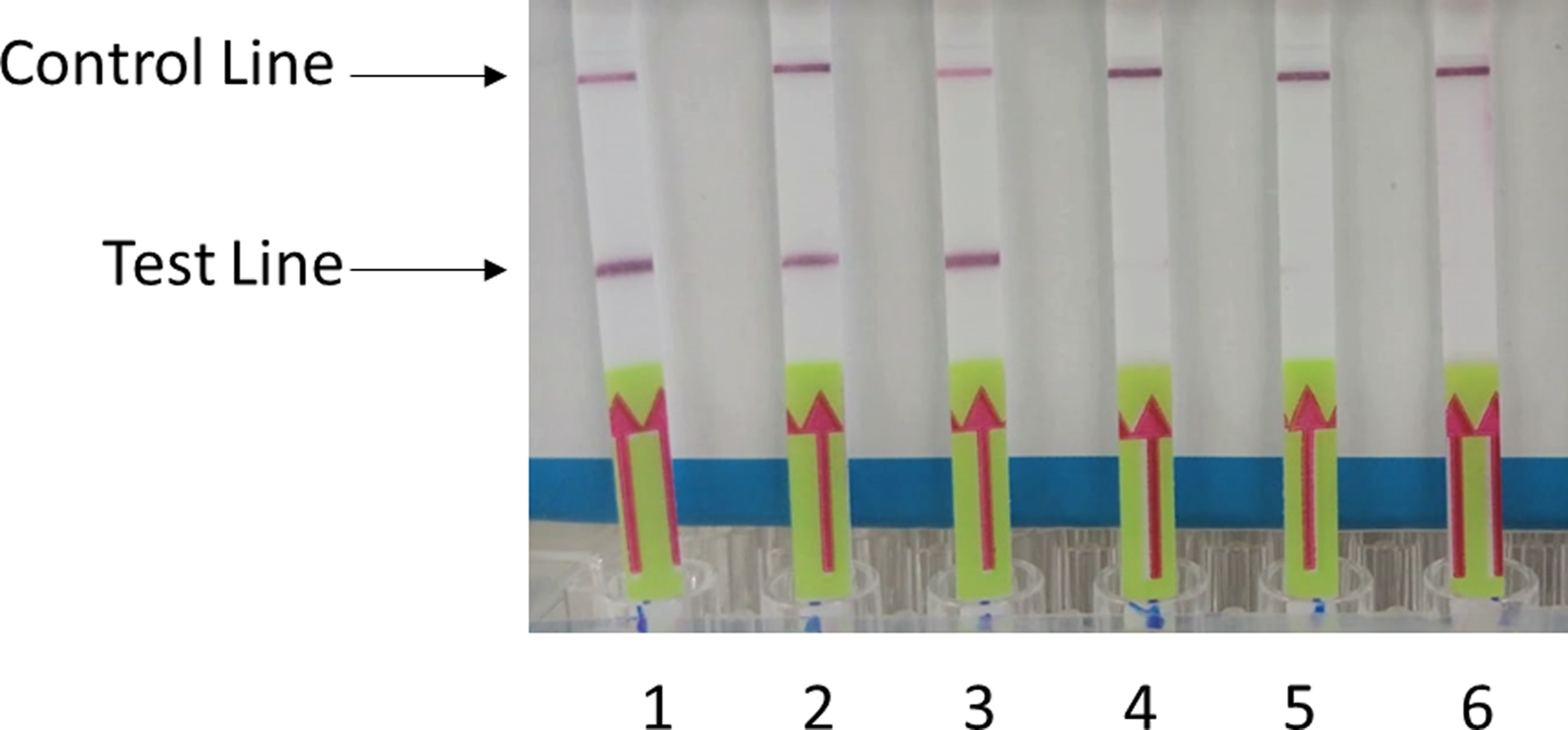

Supplement: Supplementary file 1 — Supplementary Figure 1: Optimisation of Colourimetric LAMP Assay with positive control gDNA of H. contortus. 1: 60 °C. 2: 61 °C. 3: 62 °C. 4: Non-template control. 30–45 min reaction time. Supplementary Figure 2: Colourimetric LAMP assay validation on suspected H. contortus field samples. 1: Positive Control (PEK). 2: Farm I. 3: Farm III. 4, 9 & 14: Positive control H. contortus gDNA (PEB). 5 & 6: Suspected adult H. contortus worms from deer (Farm DF). 7 & 8: Unidentified adult worms from deer large intestine (Farm DF). 10: Farm II. 12 & 13: Goat faeces sample (Farm GF). Yellow/Orange = Positive/Weak Positive, Pink/Purple = Negative. Supplementary Figure 3: Colourimetric LAMP assay validation on suspected H. contortus field samples using the individual as well as pooled faecal samples. A: Farm VI. 1-5: Individual sheep samples (random). 6: Positive control H. contortus gDNA. 7: No Template Control. B: Farm IV. 1-5: Individual sheep samples (random). 6: Positive control H. contortus gDNA. 7: Non-template control. C: Farm IV. 1-10: Pooled samples. 11: Positive control H. contortus gDNA. 12: No Template Control. D: Farm VIII. 1-3: Pooled samples. 4 & 5: Individual sheep samples (random). 6: Positive control H. contortus gDNA. 7: No Template Control. Yellow/Orange = Positive/Weak Positive, Pink/Purple = Negative. Supplementary Figure 4: LAMP Assay with H. contortus egg DNA extracted by genesig® DNA/RNA Easy Magnetic Bead Extraction (Primerdesign Ltd, UK). 1 & 2: Positive control (PEK). 4: Farm IV. 5 & 6: Farm VI. 7: Non-template Control. 8: Positive control H. contortus gDNA. Yellow/Orange = Positive/Weak Positive, Pink/Purple = Negative. Supplementary Figure 5: Bead-beating lysis and DNA extraction. Farm I samples. A: Direct Fill-FLOTAC® solution. B: Fill-FLOTAC® solution after low g centrifugation egg harvesting steps. PC: Positive control. NTC: Non-template control. Supplementary Figure 6: LF-LAMP optimisation results using diluted tagged amplicons. 1-3: Positive con [file parasite-28-82-s1.zip › parasite210006-1-olm/Supplementary Figure 6.jpg]

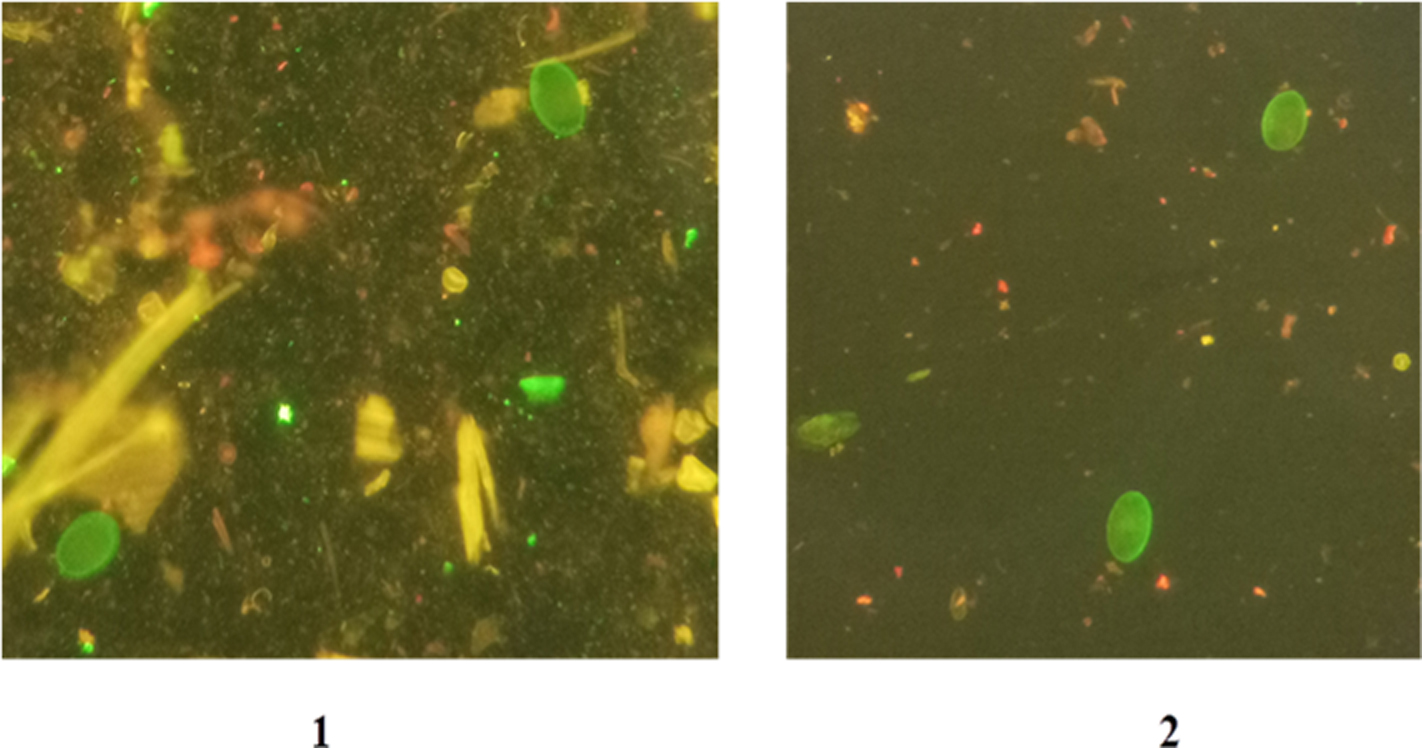

Supplement: Supplementary file 1 — Supplementary Figure 1: Optimisation of Colourimetric LAMP Assay with positive control gDNA of H. contortus. 1: 60 °C. 2: 61 °C. 3: 62 °C. 4: Non-template control. 30–45 min reaction time. Supplementary Figure 2: Colourimetric LAMP assay validation on suspected H. contortus field samples. 1: Positive Control (PEK). 2: Farm I. 3: Farm III. 4, 9 & 14: Positive control H. contortus gDNA (PEB). 5 & 6: Suspected adult H. contortus worms from deer (Farm DF). 7 & 8: Unidentified adult worms from deer large intestine (Farm DF). 10: Farm II. 12 & 13: Goat faeces sample (Farm GF). Yellow/Orange = Positive/Weak Positive, Pink/Purple = Negative. Supplementary Figure 3: Colourimetric LAMP assay validation on suspected H. contortus field samples using the individual as well as pooled faecal samples. A: Farm VI. 1-5: Individual sheep samples (random). 6: Positive control H. contortus gDNA. 7: No Template Control. B: Farm IV. 1-5: Individual sheep samples (random). 6: Positive control H. contortus gDNA. 7: Non-template control. C: Farm IV. 1-10: Pooled samples. 11: Positive control H. contortus gDNA. 12: No Template Control. D: Farm VIII. 1-3: Pooled samples. 4 & 5: Individual sheep samples (random). 6: Positive control H. contortus gDNA. 7: No Template Control. Yellow/Orange = Positive/Weak Positive, Pink/Purple = Negative. Supplementary Figure 4: LAMP Assay with H. contortus egg DNA extracted by genesig® DNA/RNA Easy Magnetic Bead Extraction (Primerdesign Ltd, UK). 1 & 2: Positive control (PEK). 4: Farm IV. 5 & 6: Farm VI. 7: Non-template Control. 8: Positive control H. contortus gDNA. Yellow/Orange = Positive/Weak Positive, Pink/Purple = Negative. Supplementary Figure 5: Bead-beating lysis and DNA extraction. Farm I samples. A: Direct Fill-FLOTAC® solution. B: Fill-FLOTAC® solution after low g centrifugation egg harvesting steps. PC: Positive control. NTC: Non-template control. Supplementary Figure 6: LF-LAMP optimisation results using diluted tagged amplicons. 1-3: Positive con [file parasite-28-82-s1.zip › parasite210006-1-olm/Supplementary Figure 7.jpg]
